# Supplementary material for: Constructive quantum interference in a bis-copper six-porphyrin nanoring
Source: Nat Commun. 2017 Mar 22;8:14842. doi: 10.1038/ncomms14842 (PMC5364408; doi:10.1038/ncomms14842)
Supplement: Supplementary Information — Supplementary Figures, Supplementary Notes and Supplementary References [file ncomms14842-s1.pdf]

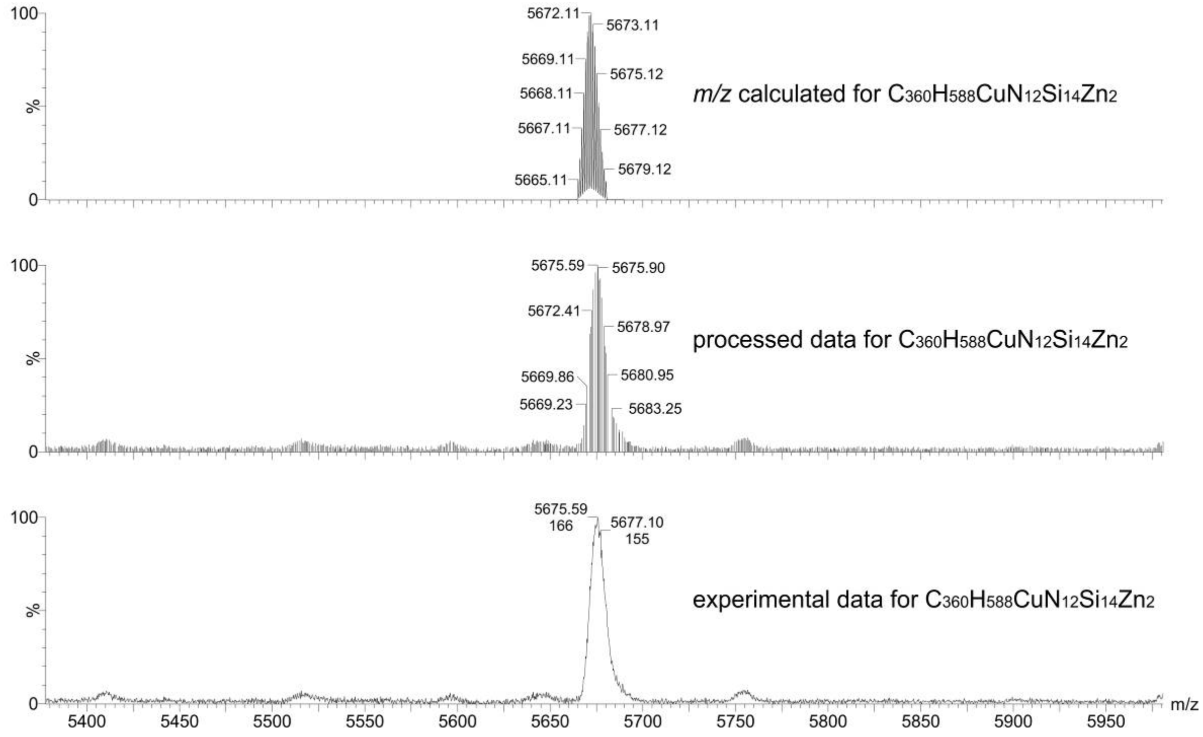

Supplementary Figure 1: MALDI-MS spectrum of $\mathbf{P3_{Cu}}$ (m/z = 5676 ($C_{360}H_{588}CuN_{12}Si_{14}Zn_2$, M^+ requires 5672), matrix: dithranol).

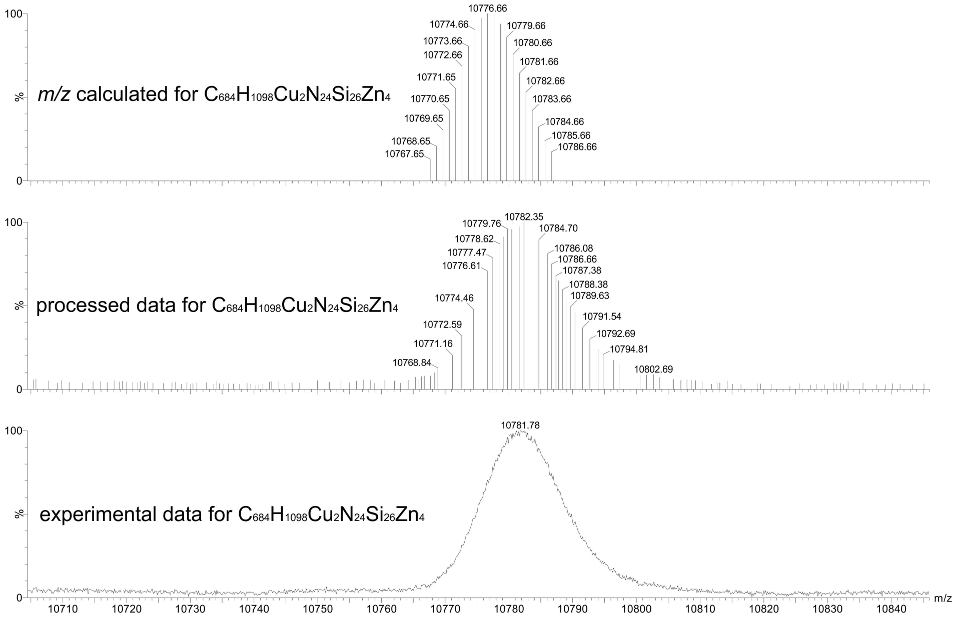

Supplementary Figure 2: MALDI-MS spectrum of $\mathbf{P6_{Cu2}}$ (m/z = 10779 ($C_{684}H_{1098}Cu_2N_{24}Si_{26}Zn_4$, M^+ requires 10777), matrix: dithranol).

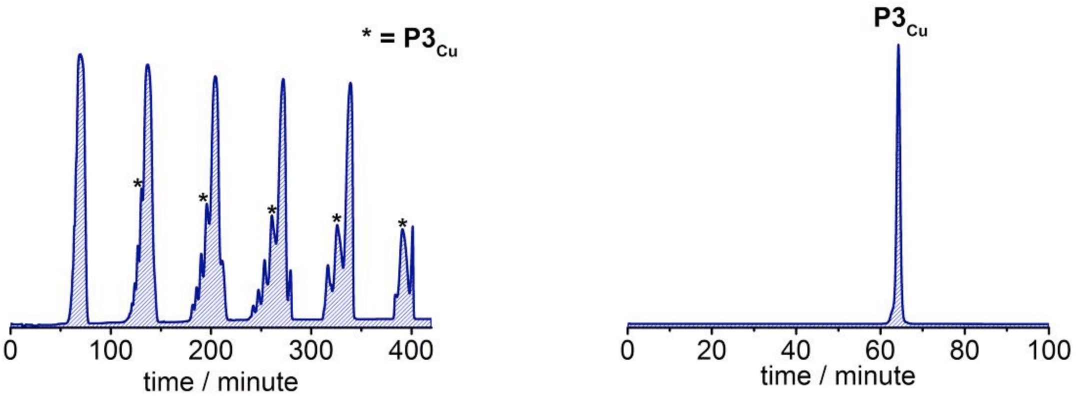

Supplementary Figure 3: Analytical recycling GPC trace of the crude reaction mixture and the purified trace of $\mathbf{P3_{Cu}}$ (eluent: 1 % pyridine in toluene).

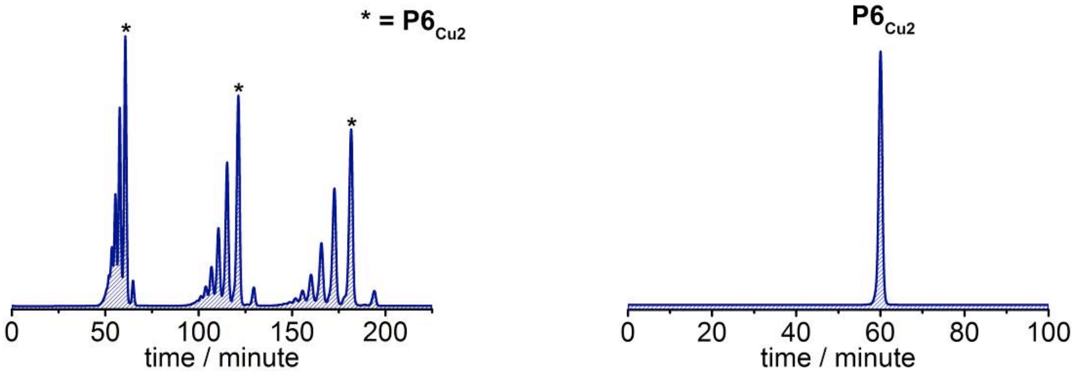

Supplementary Figure 4: Analytical recycling GPC trace of the crude reaction mixture and the purified trace of $\mathbf{P6_{Cu2}}$ (eluent: 1 % pyridine in toluene).

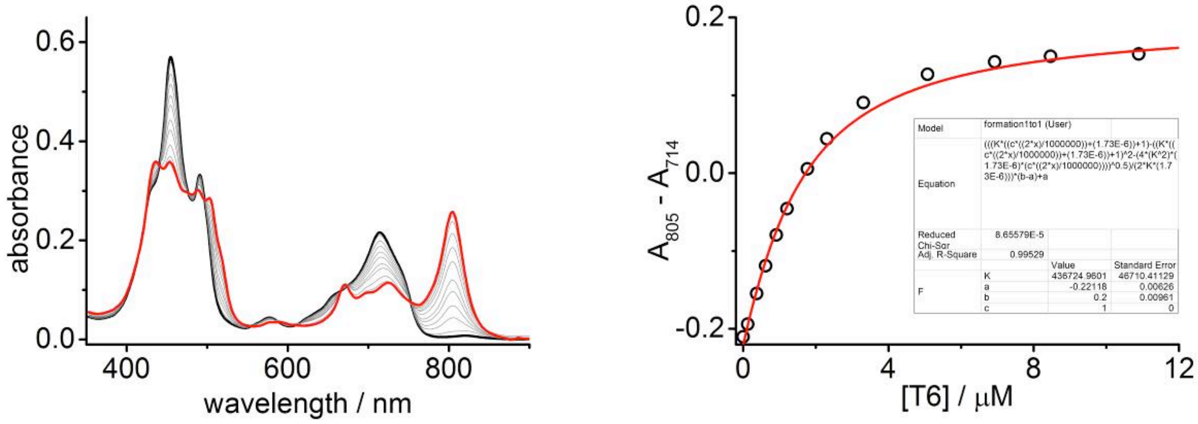

Supplementary Figure 5: UV-vis-NIR titration of $\mathbf{P3''_{Cu}}$ (1.73 μM) with **T6** in toluene at 298 K (*left*) and corresponding change in absorbance at specific wavelengths (as indicated) as a function of the concentration of **T6** (*right*). The initial and final spectra are shown in black and red, respectively. The red curve represents the fit of the binding isotherm to the experimental data (black circles) for $K = 4.37 \cdot 10^5$ M^{-1}.

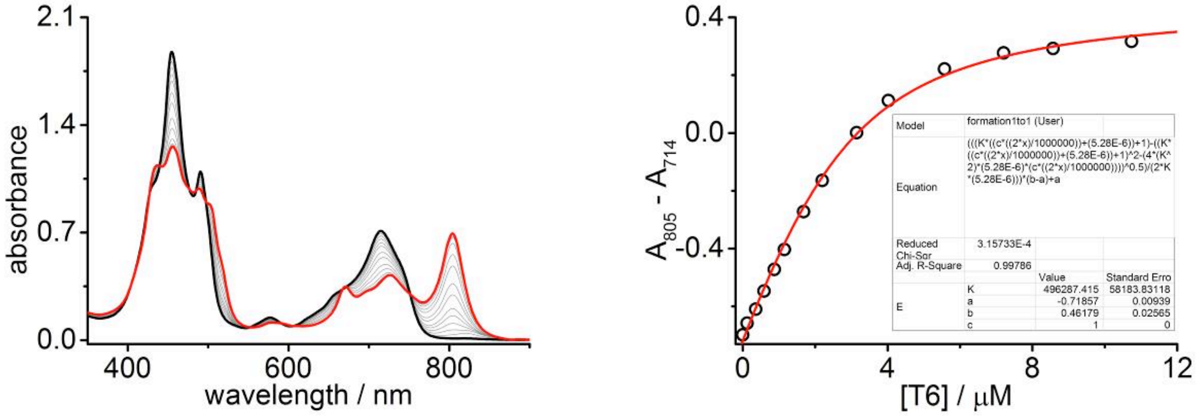

Supplementary Figure 6: UV-vis-NIR titration of $\mathbf{P3''_{Cu}}$ (5.28 μM) with **T6** in toluene at 298 K (*left*) and corresponding change in absorbance at specific wavelengths (as indicated) as a function of the concentration of **T6** (*right*). The initial and final spectra are shown in black and red, respectively. The red curve represents the fit of the binding isotherm to the experimental data (black circles) for $K = 4.96 \cdot 10^5$ M^{-1}.

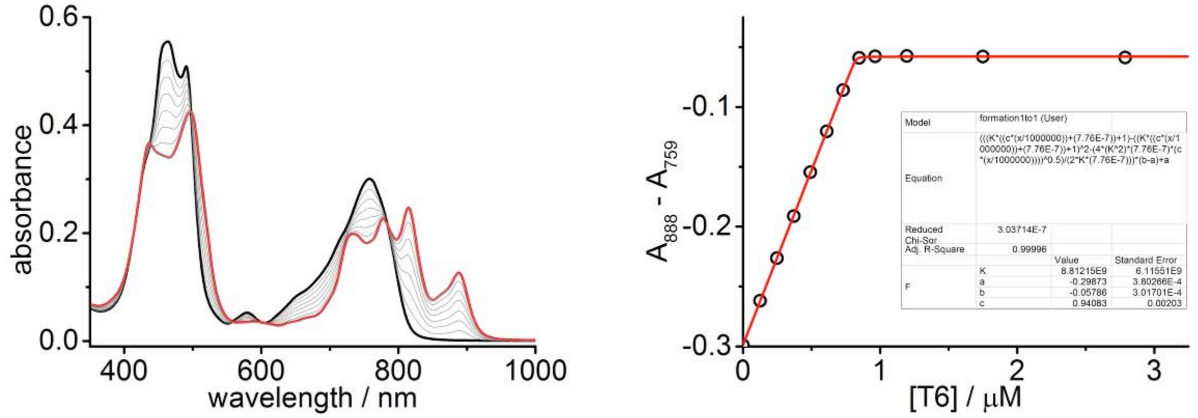

Supplementary Figure 7: UV-vis-NIR titration of $\mathbf{P6''_{Cu2}}$ (0.78 μM) with **T6** in toluene at 298 K (*left*) and corresponding change in absorbance at specific wavelengths (as indicated) as a function of the concentration of **T6** (*right*). The initial and final spectra are shown in black and red, respectively. The red curve represents the fit of the binding isotherm to the experimental data (black circles) for $K = 8.81 \cdot 10^9$ M^{-1}.

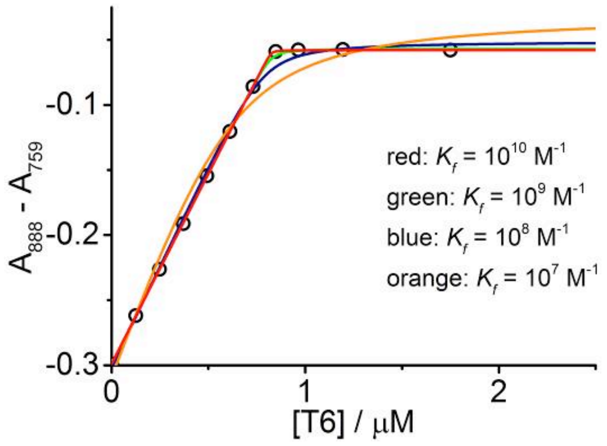

Supplementary Figure 8: Evaluation of the magnitude of the binding constant between $\mathbf{P6''_{Cu2}}$ and **T6**. Fits to the experimental data are shown for four (fixed) values of the binding constant K_f. The other parameters entering the simulation were allowed to vary.

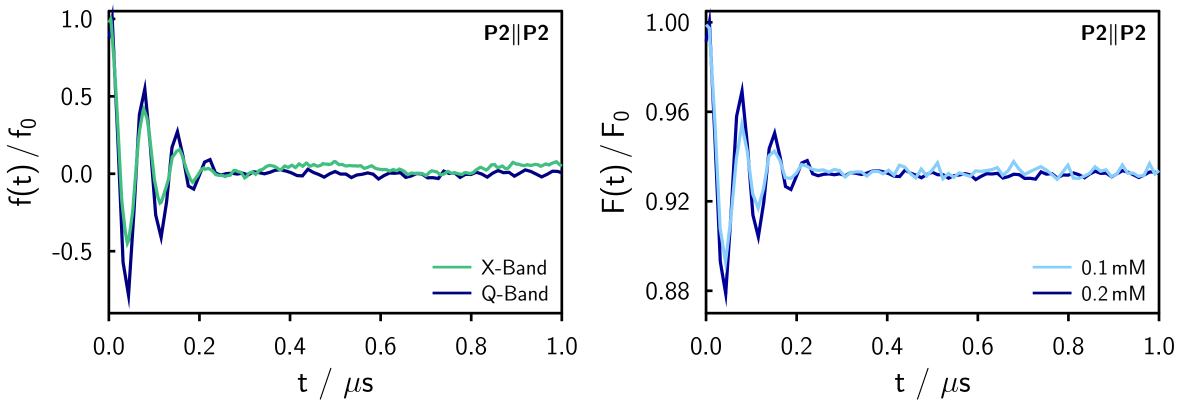

Supplementary Figure 9: Comparison of the background corrected DEER traces for **P2||P2** measured at X- and Q-band (*left*) and comparison of the traces recorded and Q-band at two different concentrations (*right*).

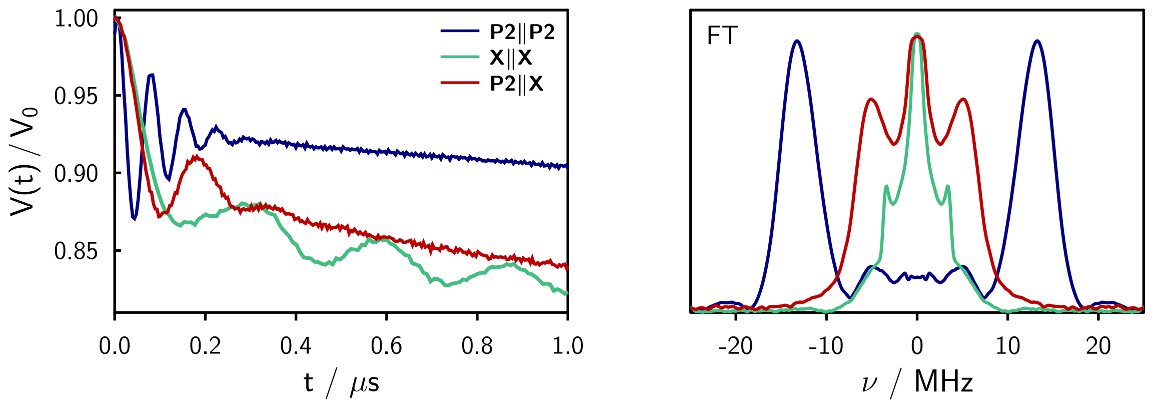

Supplementary Figure 10: Phase-corrected experimental DEER traces for the three investigated samples (*left*) and Fourier transformations of the background-corrected traces shown in the main text (*right*).

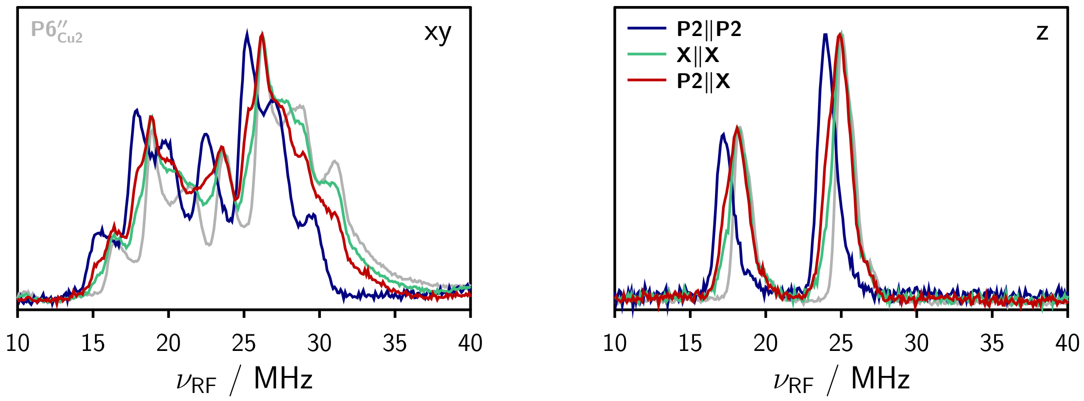

Supplementary Figure 11: Davies ENDOR spectra for the three samples recorded at Q-band at field positions corresponding to the *xy* (*left*) and *z* (*right*) orientations, respectively. For comparison the spectrum of the linear hexamer without template, $\mathbf{P6''_{Cu2}}$, is shown in the background in gray.

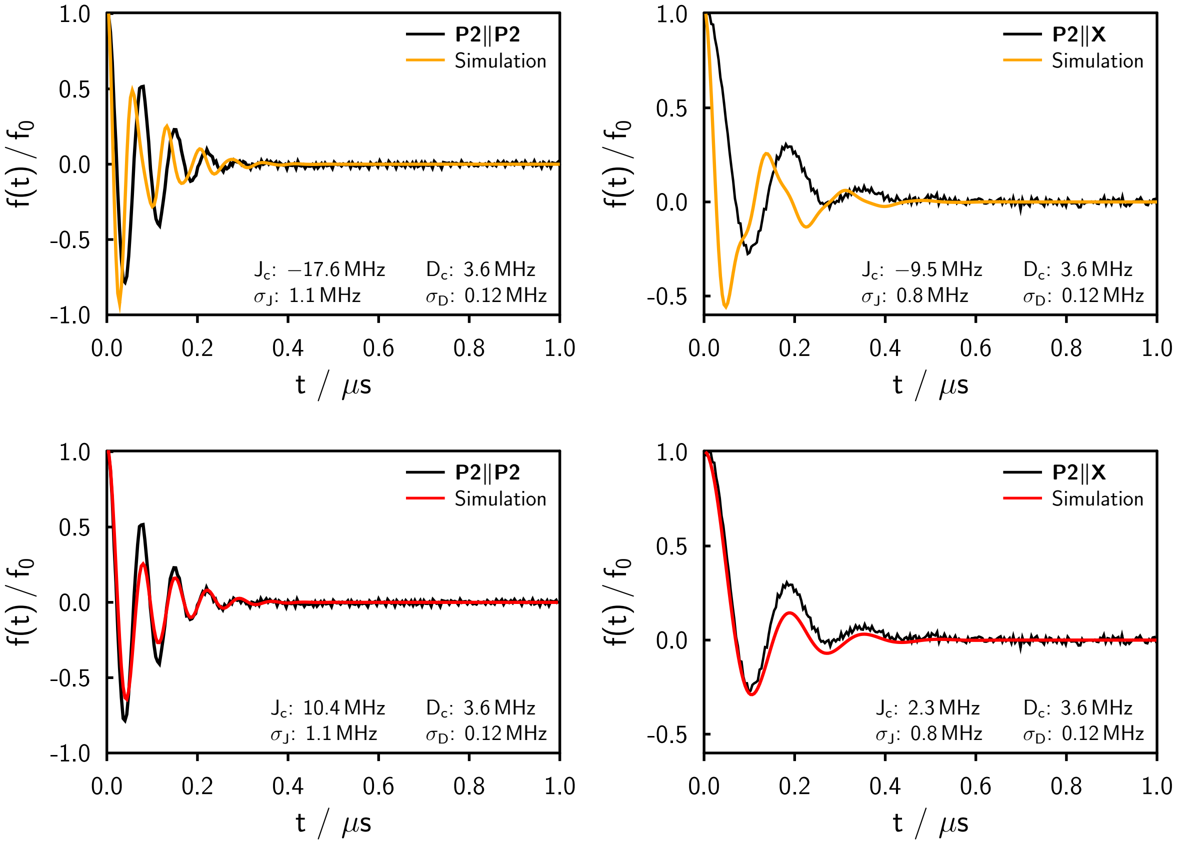

Supplementary Figure 12: Simulation of the form factor of the background-corrected DEER trace for **P2**||**P2** (*left*) and **P2**||**X** (*right*). The traces simulated for a negative J value (*top*, orange) and a positive J value (*bottom*, red) are contrasted. The fit is clearly worse for a negative J and cannot be significantly improved when varying J without changing its sign, confirming the choice of a positive sign for J.

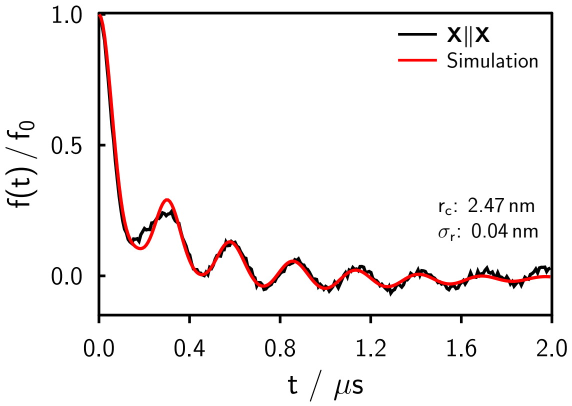

Supplementary Figure 13: Simulation of the form factor of the background-corrected DEER trace for **X**||**X** assuming a distance distribution consisting of two components with Gaussian distribution.

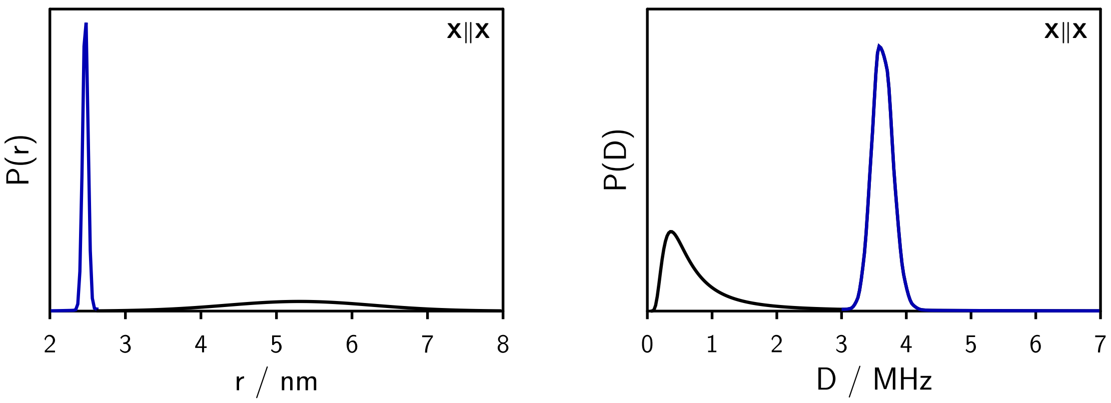

Supplementary Figure 14: Distance distribution ($g = 2.05$) resulting from a least-squares fit of the simulation to the experimental data for **X**||**X** when allowing the center and width of a second Gaussian to vary (*left*) and corresponding frequency distribution (*right*).

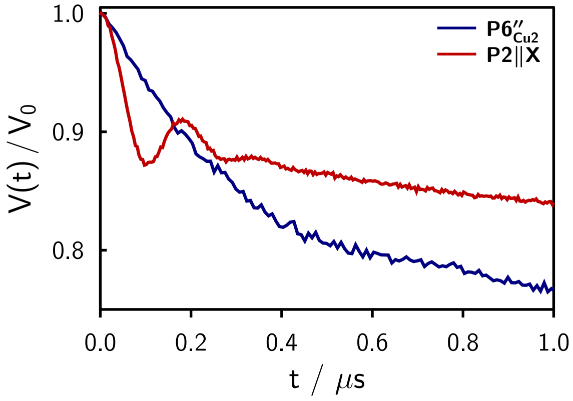

Supplementary Figure 15: Comparison of the DEER traces before background correction of $\mathbf{P6''_{Cu2}}$ and **P2**||**X**.

Supplementary Note 1: Synthesis and Characterization

The synthesis of the compound referred to in the main text of the manuscript as **P2||P2** was already published elsewhere [1]. For a more precise description of the synthetic procedures, the compound **P2||X** will be referred to as $\mathbf{P6''_{Cu2} \cdot T6}$ for a linear porphyrin hexamer with two copper units ($\mathbf{P6_{Cu2}}$) bound to a hexapyridyl (**T6**) template. Analogously, **X||X** is referred to as $\mathbf{(P3''_{Cu})_2 \cdot T6}$.

Synthetic and Experimental Procedures

Dry toluene and THF were obtained by passing the solvents through columns of alumina, under nitrogen. Diisopropylamine (DIPA) was distilled from CaH_2 and kept over activated molecular sieves (3 Å, 8–12 mesh). Unless specified otherwise, all other solvents were used as commercially supplied. Flash chromatography was carried out on silica gel 60 under positive pressure, whereas analytical thin-layer chromatography was carried out on aluminum-backed silica gel 60 F254 plates. Visualization was achieved using UV light when necessary.

All UV-vis-NIR spectra were recorded in solution using a Perkin-Lambda 20 spectrometer (1 cm path length quartz cell).

Unless stated otherwise, $^{1}H/^{13}C$ NMR spectra were recorded at 298 K using a Bruker AV400 (400/100 MHz) instrument. ^{1}H, and ^{13}C NMR spectra are reported in ppm; coupling constants are given in Hertz, to the nearest 0.1 Hz. The solvents used were $CDCl_3$ or a mixture of $CDCl_3$ and pyridine-d_5 (99:1 by volume). The presence of the paramagnetic Cu(II) centers in some of the oligomers drastically complicates the interpretation of the ^{1}H NMR spectra. Many signals cannot be observed at all due to shortening of the relaxation times. Some signals can be observed but, due to broadening, cannot be accurately integrated and therefore the calculated integration, rather than the experimentally observed integrations, will be reported below.

MALDI-TOF mass spectra were carried out using a Waters MALDI Micro MX spectrometer.

Synthesis of $\mathbf{P1_{2H}}$

$\mathbf{P1_{Zn}}$ (280 mg, 0.12 mmol) was dissolved in $CHCl_3$ (40 mL). Trifluoroacetic acid (0.47 mL) was mixed with $CHCl_3$ (5 mL) to give a 10 % solution. The TFA solution was added dropwise to the porphyrin solution and the reaction mixture was stirred at room temperature for 15 minutes. The reaction mixture was passed through a short plug of silica gel ($CHCl_3$) to give the title compound as a dark oil (260 mg, 96 %).

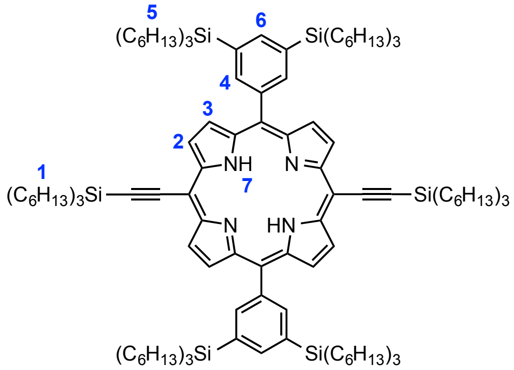

^{1}H NMR (400 MHz, $CDCl_3$, 298 K): δ_H (ppm) 9.61 (4H, d, J = 4.7 Hz, H2), 8.78 (4H, d, J = 4.7 Hz, H3), 8.24 (4H, m, H4), 8.00 (2H, m, H6), 1.80–0.82 (234H, m, H1/H5), −2.11 (2H, s, H7).

MALDI-TOF: m/z = 2207 ($C_{144}H_{250}N_4Si_6$, M^+ requires 2206).

Synthesis of $\mathbf{P1_{Cu}}$

$\mathbf{P1_{2H}}$ (150 mg, 0.068 mmol) was dissolved in $CHCl_3$ (30 mL). $Cu(OAc)_2 \cdot H_2O$ (494 mg, 2.72 mmol) was added and the reaction mixture was stirred at 60 °C for 15 minutes. The mixture was allowed to cool to room temperature after which it was passed through a short plug of silica gel ($CHCl_3$) to give the title compound as a dark oil (110 mg, 71 %).

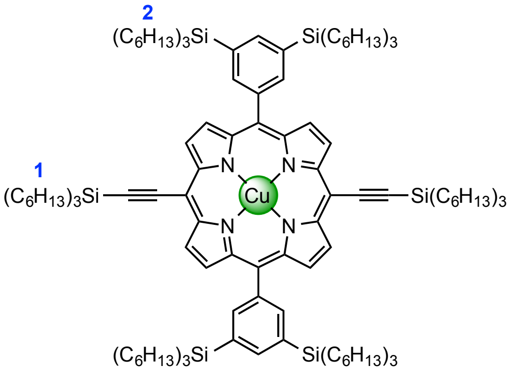

^{1}H NMR (400 MHz, $CDCl_3$, 298 K): δ_H (ppm) broad, 1.80−0.61 (234H, m, H1/H2).

MALDI-TOF: m/z = 2267 ($C_{144}H_{248}CuN_4Si_6$, M^+ requires 2266).

$\lambda_{max}(CHCl_3)$ / nm log(ε) : 434 (5.58), 567 (4.20), 610 (4.53).

Synthesis of $\mathbf{P1'_{Cu}}$

$\mathbf{P1_{Cu}}$ (131 mg, 0.057 mmol) was dissolved in $CHCl_3$ (3.5 mL) and CH_2Cl_2 (13 mL). Tetra-*n*-butylammonium fluoride (86 μL, 1.0 M solution in THF, 0.086 μmol) was added to the stirred solution. The progress of the reaction was monitored by TLC (petrol ether) until an optimal mixture was reached. The mixture was immediately passed through a short plug of silica gel ($CHCl_3$) to give the title compound as a purple oil (48 mg, 42 %).

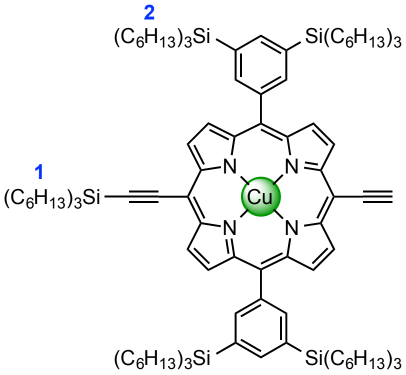

^{1}H NMR (400 MHz, $CDCl_3$, 298 K): δ_H (ppm) broad, 1.70−0.68 (195H, m, H1/H2).

MALDI-TOF: m/z = 1986 ($C_{126}H_{210}CuN_4Si_5$, M^+ requires 1985).

Synthesis of $\mathbf{P1''_{Cu}}$

$\mathbf{P1_{Cu}}$ (110 mg, 0.049 mmol) was dissolved in CH_2Cl_2 (15 mL). Tetra-*n*-butylammonium fluoride (0.97 mL, 1.0 M solution in THF, 0.97 mmol) was added dropwise and the reaction mixture was stirred at room temperature for 15 minutes. The reaction mixture was immediately passed through a short plug of silica gel ($CHCl_3$) to give the title compound as a purple oil (80 mg, 97 %).

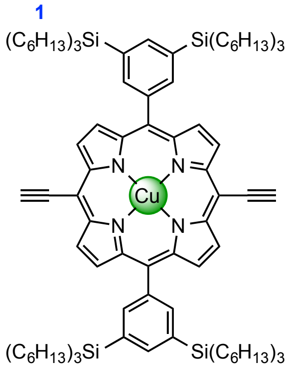

^{1}H NMR (400 MHz, $CDCl_3$, 298 K): δ_H (ppm) broad, 1.72−0.70 (156H, m, H1).

MALDI-TOF: m/z = 1702 ($C_{108}H_{172}CuN_4Si_4$, M^+ requires 1702).

Synthesis of $\mathbf{P3_{Cu}}$

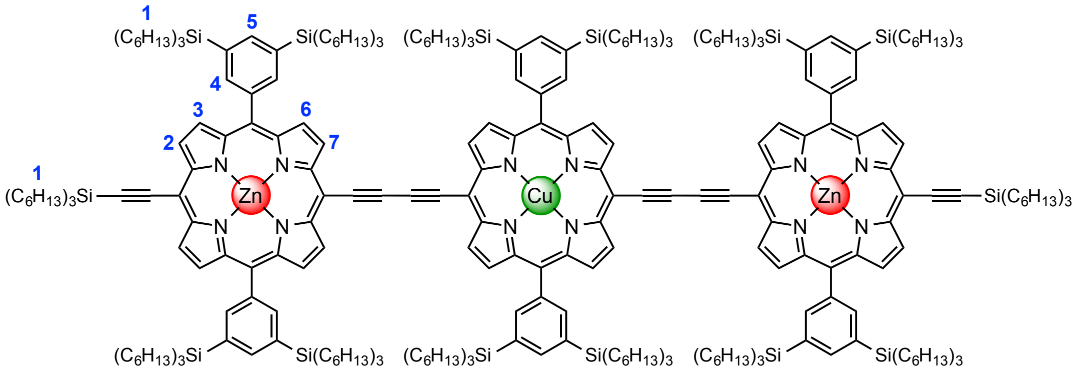

$\mathbf{P1''_{Cu}}$ (410 mg, 0.21 mmol) and $\mathbf{P1'_{Zn}}$ (70 mg, 0.04 mmol) were dissolved in $CHCl_3$ (25 mL). A catalyst solution was prepared by dissolving $PdCl_2(PPh_3)_2$ (21.7 mg, 0.03 mmol), CuI (21.6 mg, 0.11 mmol) and 1,4-benzoquinone (63.8 mg, 0.59 mmol) in $CHCl_3$ (14 mL) and diisopropylamine (0.7 mL). The catalyst solution was added to the porphyrin solution and the reaction mixture was stirred at room temperature for 1 hour. The crude reaction mixture was passed through a short plug of silica gel (1 % pyridine in $CHCl_3$), passed over a SEC column (1 % pyridine in $CHCl_3$), and finally the oligomers were separated by recycling GPC (1 % pyridine in toluene) to give $\mathbf{P3_{Cu}}$ as a dark oily substance (77.5 mg, 34 %).

^{1}H NMR (400 MHz, $CDCl_3$, 298 K): δ_H (ppm) 9.77 (4H, m, H7), 9.67 (4H, d, J = 4.3 Hz, H2), 8.91 (4H, m, H6), 8.84 (4H, d, J = 4.3 Hz, H3), 8.25 (8H, m, H4), 8.00 (4H, m, H5), 1.81−0.77 (546H, m, H1).

MALDI-TOF: m/z = 5676 ($C_{360}H_{588}CuN_{12}Si_{14}Zn_2$, M^+ requires 5672).

λ_{max}($CHCl_3$) / nm log(ε) : 457 (5.69), 488 (3.79), 579 (4.16), 669 (2.80), 726 (1.93).

Synthesis of $\mathbf{P3'_{Cu}}$

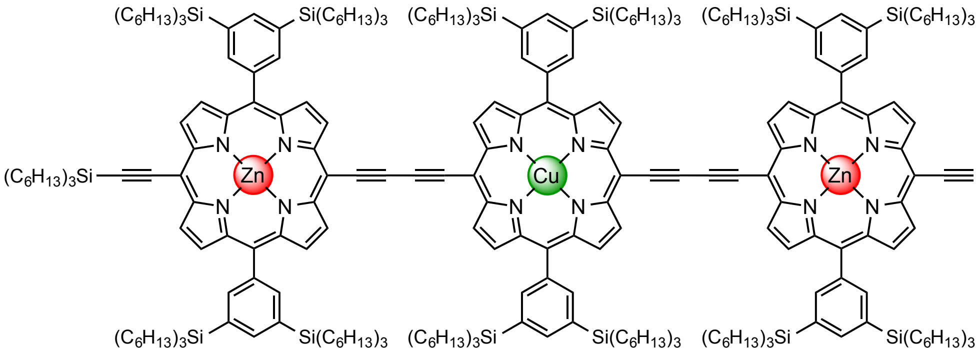

$\mathbf{P3_{Cu}}$ (77.5 mg, 13.7 μmol) was dissolved in CH_2Cl_2 (4.5 mL) and $CHCl_3$ (4.5 mL). Tetra-*n*-butylammonium fluoride (20.5 μL, 1.0 M solution in THF, 20.5 μmol) was added to the stirred solution. The progress of the reaction was monitored by TLC (hexane:ethyl acetate:pyridine = 90:5:5) until an optimal mixture was reached. The mixture was immediately passed through a short plug of silica gel (1 % pyridine in $CHCl_3$). We were unable to purify the reaction mixture further and the

fraction containing the desired product was concentrated to dryness and used in the next step to form the hexamer, **P6$_{Cu2}$**, without further purification, assuming that half of the starting material was converted to the title compound.

Synthesis of P3$''_{Cu}$

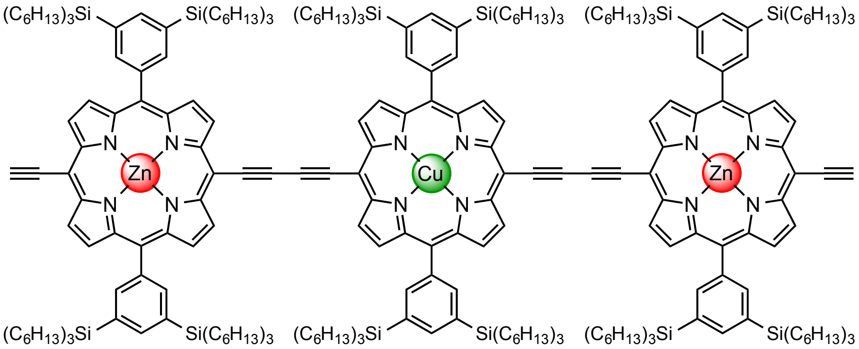

P3$_{Cu}$ (6 mg, 1.2 μmol) was dissolved in CH_2Cl_2 (1.5 mL). Tetra-*n*-butylammonium fluoride (24 μL, 1.0 M solution in THF, 24 μmol) was added and the reaction mixture was stirred at room temperature for 15 minutes. The reaction mixture was immediately passed through a short plug of silica gel ($CHCl_3$) to give the title compound as a dark oil (5.2 mg, 96 %).

MALDI-TOF: m/z = 5106 ($C_{324}H_{512}CuN_{12}Si_{12}Zn_2$, M^+ requires 5105).

λ_{max}($CHCl_3$) / nm log(ε) : 454 (5.55), 487 (5.21), 577 (4.38), 720 (5.08).

Synthesis of P6$_{Cu2}$

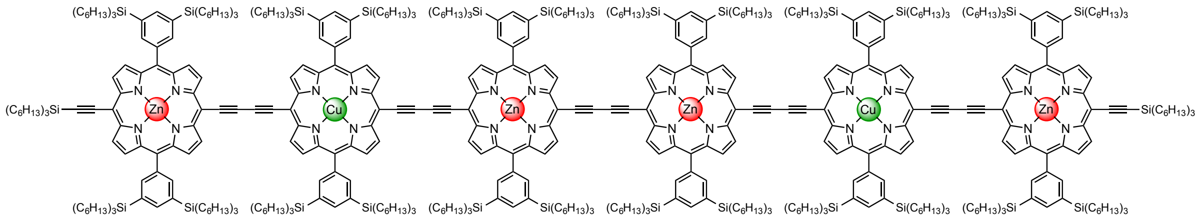

P3$'_{Cu}$ (35 mg, 6.5 μmol) was dissolved in toluene (1 mL). $PdCl_2(PPh_3)_2$ (0.23 mg, 0.32 μmol), CuI (0.62 mg, 0.32 μmol) and 1,4-benzoquinone (1.40 mg, 13.0 μmol) were dissolved in toluene (0.3 mL) and diisopropylamine (0.3 mL). The catalyst solution was immediately added and the mixture was stirred at room temperature for 1 hour. The crude reaction mixture was passed through a short plug of silica gel (1 % pyridine in $CHCl_3$), passed over a SEC column (1 % pyridine in $CHCl_3$), and finally the oligomers were separated by recycling GPC (1 % pyridine in toluene) to give **P6$_{Cu2}$** as a dark oily substance (15.1 mg, 43 %).

^{1}H NMR (400 MHz, $CDCl_3$, 298 K): δ_H (ppm) 9.90 (8H, m, β-H), 9.67 (8H, m, β-H), 8.96 (8H, m, β-H), 8.85 (8H, m, β-H), 8.31 (8H, m, ortho-aryl-H), 8.25 (8H, m, ortho-aryl-H), 8.02 (4H, m, para-aryl-H), 8.00 (4H, m, para-aryl-H), 1.82−0.79 (1014H, m, THS-H).

MALDI-TOF: m/z = 10779 ($C_{584}H_{1098}Cu_2N_{24}Si_{26}Zn_4$, M^+ requires 10777).

λ_{max}($CHCl_3$) / nm log(ε) : 458 (5.88), 488 (5.78), 581 (4.76), 663 (5.07), 766 (5.54).

Synthesis of $\mathbf{P6''_{Cu2}}$

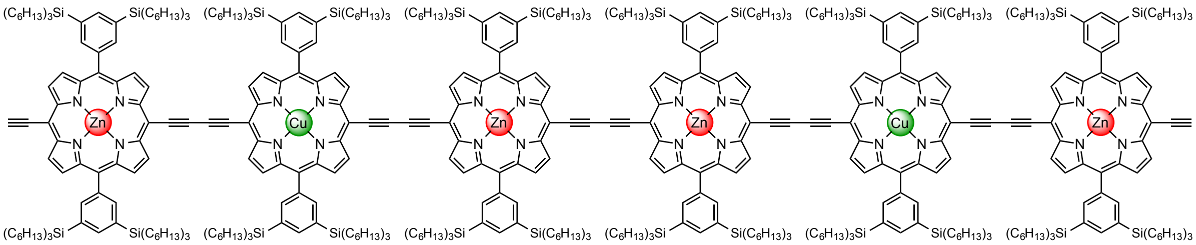

$\mathbf{P6_{Cu2}}$ (9.0 mg, 0.88 μmol) was dissolved in CH_2Cl_2 (1 mL). Tetra-*n*-butylammonium fluoride (18 μL, 1.0 M solution in THF, 18 μmol) was added and the reaction mixture was stirred at room temperature for 15 minutes. The crude mixture was immediately passed through a short plug of silica gel ($CHCl_3$) to give the title compound as a dark oil (6.5 mg, 76 %).

MALDI-TOF: m/z = 10207 ($C_{648}H_{1022}Cu_2N_{24}Si_{24}Zn_4$, M^+ requires 10212).

$\boldsymbol{\lambda_{max}}$**($CHCl_3$) / nm log($\varepsilon$) :** 455 (6.09), 488 (6.01), 579 (5.00), 659 (5.26), 766 (5.76).

Proof of Purity

To prove the purity of the relevant compounds, clean MALDI-MS spectra and clean analytical GPC traces are provided for $\mathbf{P3_{Cu}}$ and $\mathbf{P6_{Cu2}}$ in **Supplementary Figures 1 to 4**.

Supplementary Note 2: Binding Studies

To monitor complex formation and to characterize the accompanying spectral changes, UV-vis-NIR titrations of toluene solutions of $\mathbf{P3''_{Cu}}$ and $\mathbf{P6''_{Cu2}}$ with **T6** were carried out at room temperature (298 K). Since it was shown before that the presence of copper centers in the structures can hinder complex formation [1], it might be expected that complex formation is not complete after addition of the theoretically expected stoichiometric equivalents of **T6** of 0.5 and 1 for $\mathbf{P3''_{Cu}}$ and $\mathbf{P6''_{Cu2}}$, respectively.

For all titrations, the spectral changes at characteristic wavelengths were monitored as a function of the template concentration. Complex formation is characterized by a red-shift of the porphyrin Q-band and the disappearance of the central peak of the porphyrin Soret band around 450 nm [1]. The results for $\mathbf{P3''_{Cu}}$ and $\mathbf{P6''_{Cu2}}$ are shown in **Supplementary Figures 5 to 7**. While a 1:1 complex seems to form between $\mathbf{P6''_{Cu2}}$ and **T6** at room temperature, it is noted that the formation of $\mathbf{(P3''_{Cu})_2 \cdot T6}$ (= **X**||**X**) is far from being complete after addition of 0.5 equivalents of **T6**.

To quantify the binding constant (K) for complex formation between the linear copper-containing porphyrin oligomers and the hexapyridyl template **T6**, a 1:1 binding isotherm was fit to the experimental data shown in **Supplementary Figures 5 to 7**. The relative change in absorbance is given as

$$\frac{A - A_{\text{initial}}}{A_\infty - A_{\text{initial}}} = \left(\frac{(K([L]+[P]_0)+1) - \sqrt{(K([L]+[P]_0)+1)^2 - 4K^2[P]_0[L]}}{2K[P]_0} \right) \quad \text{(S1)}$$

where A is the measured absorbance at a specific wavelength or the difference in absorbance between two wavelengths, A_{initial} and A_∞ are the initial and asymptotic final absorbance at the chosen wavelength, respectively, K is the association constant between ligand and porphyrin host, and $[L]$ and $[P]_0$ are the concentrations of ligand and porphyrin host, respectively. In the fitting procedure, the three parameters A_{initial}, A_∞, and K were allowed to vary.

The binding of $\mathbf{P3''_{Cu}}$ to **T6** is found to be weak enough so that the data can reliably be fit assuming a 1:1 binding isotherm (**Equation (S1)**). The two $\mathbf{P3''_{Cu}}$ units bind independently to the **T6** template with an association constant of $K = 4.7 \pm 0.4 \cdot 10^5\,\text{M}^{-1}$. At the concentrations employed in the EPR experiments ($2 \cdot 10^{-4}$ M) the probability for a porphyrin trimer to be bound to **T6** is about 90 %, suggesting a mole fraction for the 2:1 complex of about 0.8 at 298 K.

The analysis of the data for the binding of $\mathbf{P6''_{Cu2}}$ to **T6** reveals a binding constant of $K = 8.81 \cdot 10^9\,\text{M}^{-1}$. Since the binding is too strong for the fit to be reliable, further simulations were carried out for fixed values of K. The results are shown in **Supplementary Figure 8** and allow to conclude that the binding constant is greater than $10^8\,\text{M}^{-1}$, suggesting complete complex formation at 298 K.

The results thus indicate that in the case of the **X||X** sample for the EPR measurements, prepared at a $\mathbf{P3''_{Cu}}$:**T6** ratio of 2:1, only about 80 % of **T6** templates will have two of the trimers bound. The **X||X** sample is most likely a mixture of unbound $\mathbf{P3''_{Cu}}$, $\mathbf{P3''_{Cu} \cdot T6}$, free **T6**, $\mathbf{(P3''_{Cu})_2 \cdot T6}$ and possibly other complexes involving more than one **T6** template.

Most of the hypothesized contributions in the EPR sample referred to as **X||X** do not affect the DEER measurements. Unbound $\mathbf{P3''_{Cu}}$, $\mathbf{P3''_{Cu} \cdot T6}$, and free **T6** should not contribute to the DEER signal. However any type of complexes involving more than one **T6** template and more than one $\mathbf{P3''_{Cu}}$ molecule could potentially lead to the observation of additional distances as will be further discussed below.

Supplementary Note 3: Additional Experimental Details for EPR

For information on the preparation of the EPR samples and settings used in the DEER experiments, the reader is referred to the method section of the manuscript.

Davies ENDOR measurements at 15 K were carried out at Q-band frequencies (34.0 GHz) on a Bruker ELEXSYS E580 pulsed EPR spectrometer equipped with a Bruker EN 5107D2 resonator and an Oxford Instruments liquid helium flow cryostat (CF935) in order to determine the nitrogen hyperfine and nuclear quadrupole couplings. All experimental ENDOR spectra were obtained using the pulse sequence $\pi - T - \pi/2 - \tau - \pi - \tau -$ echo with $\pi/2$ and π pulse lengths of 20 and 40 ns, respectively, and an evolution period T of 27 μs during which a 25 μs RF π pulse was applied. The inter-pulse delay τ for the detection sequence was chosen to be 220 ns. The whole echo signal was integrated and recorded as a function of the RF frequency in stochastic mode with an RF increment of 0.1 MHz. The RF power was adjusted based on a nutation experiment.

Supplementary Note 4: Derivation of the Analytic Expression for DEER Including D and J

A two electron system with dipole-dipole and exchange coupling has the the following Hamiltonian:

$$\begin{aligned}\hat{\mathcal{H}}(\Omega) = {} & \omega_1 \hat{\mathcal{L}}_Z^{(1)} + \omega_2 \hat{\mathcal{L}}_Z^{(2)} - \sqrt{6}\,\frac{\mu_0}{4\pi}\,\frac{\gamma_1\gamma_2\hbar}{r^3} \sum_{m=-2}^{2} \hat{\mathcal{T}}_{2,m} D_{m,0}^{(2)}(\Omega) \\ & + J\left(\hat{\mathcal{L}}_X^{(1)}\hat{\mathcal{L}}_X^{(2)} + \hat{\mathcal{L}}_Y^{(1)}\hat{\mathcal{L}}_Y^{(2)} + \hat{\mathcal{L}}_Z^{(1)}\hat{\mathcal{L}}_Z^{(2)}\right)\end{aligned} \tag{S2}$$

where Ω represents a set of three Euler angles, $D_{m,0}^{(2)}(\Omega)$ are second-rank Wigner D functions, J is defined in the NMR convention in angular frequency units and the irreducible spherical tensor operators are:

$$\begin{aligned}\hat{\mathcal{T}}_{2,2} &= +\frac{1}{2}\hat{\mathcal{L}}_+^{(1)}\hat{\mathcal{L}}_+^{(2)} \\ \hat{\mathcal{T}}_{2,1} &= -\frac{1}{2}\left(\hat{\mathcal{L}}_Z^{(1)}\hat{\mathcal{L}}_+^{(2)} + \hat{\mathcal{L}}_+^{(1)}\hat{\mathcal{L}}_Z^{(2)}\right) \\ \hat{\mathcal{T}}_{2,0} &= +\sqrt{\frac{2}{3}}\left(\hat{\mathcal{L}}_Z^{(1)}\hat{\mathcal{L}}_Z^{(2)} - \frac{1}{4}\left(\hat{\mathcal{L}}_+^{(1)}\hat{\mathcal{L}}_-^{(2)} + \hat{\mathcal{L}}_-^{(1)}\hat{\mathcal{L}}_+^{(2)}\right)\right) \\ \hat{\mathcal{T}}_{2,-1} &= +\frac{1}{2}\left(\hat{\mathcal{L}}_Z^{(1)}\hat{\mathcal{L}}_-^{(2)} + \hat{\mathcal{L}}_-^{(1)}\hat{\mathcal{L}}_Z^{(2)}\right) \\ \hat{\mathcal{T}}_{2,-2} &= +\frac{1}{2}\hat{\mathcal{L}}_-^{(1)}\hat{\mathcal{L}}_-^{(2)}\end{aligned} \tag{S3}$$

Applying the rotating frame with respect to the Zeeman Hamiltonian

$$\hat{\mathcal{H}}_0 = \omega_1 \hat{\mathcal{L}}_Z^{(1)} + \omega_2 \hat{\mathcal{L}}_Z^{(2)} \tag{S4}$$

and relying on the fact that the spin echo would refocus the g-factor offset variations, we obtain the following rotating frame Hamiltonian in the limit when $|\omega_1 - \omega_2| \gg |D| + |J|$ (high field and weak coupling approximations):

$$\begin{aligned}\hat{\mathcal{H}}(\Omega) &= \frac{\mu_0}{4\pi}\,\frac{\gamma_1\gamma_2\hbar}{r^3}\left(1 - 3\cos^2\theta\right)\,\hat{\mathcal{L}}_Z^{(1)}\hat{\mathcal{L}}_Z^{(2)} + J\hat{\mathcal{L}}_Z^{(1)}\hat{\mathcal{L}}_Z^{(2)} \\ &= D\left(1 - 3\cos^2\theta\right)\,\hat{\mathcal{L}}_Z^{(1)}\hat{\mathcal{L}}_Z^{(2)} + J\hat{\mathcal{L}}_Z^{(1)}\hat{\mathcal{L}}_Z^{(2)}\end{aligned} \tag{S5}$$

which, when acting on the transverse magnetisation, would produce the following oscillation:

$$s(\theta, t) = \cos\left(\left[D\left(1 - 3\cos^2\theta\right) + J\right]t\right) \tag{S6}$$

Averaging this over all molecular orientations yields:

$$\begin{aligned} f(t) &= \frac{1}{2} \int_0^{\pi} \cos\left(\left[D\left(1-3\cos^2\theta\right)+J\right] t\right) \sin\theta \, \mathrm{d}\theta \\ &= \sqrt{\frac{\pi}{6\,D\,t}} \left[\cos\left(\left(D+J\right)t\right) \mathrm{FrC}\left(\sqrt{\frac{6\,D\,t}{\pi}}\right) + \sin\left(\left(D+J\right)t\right) \mathrm{FrS}\left(\sqrt{\frac{6\,D\,t}{\pi}}\right)\right] \end{aligned} \tag{S7}$$

with the Fresnel functions defined according to:

$$\mathrm{FrC}(x) = \int_0^x \cos\left(t^2\right)\,\mathrm{d}t \qquad \mathrm{FrS}(x) = \int_0^x \sin\left(t^2\right)\,\mathrm{d}t \tag{S8}$$

In these equations, D and J are given in units of $\frac{\mathrm{rad}}{\mathrm{s}}$.

The use of this equation requires orientation selective effects on the modulation frequency to be negligible. In the present case both detection and pump pulses were placed in the $g_\perp$ region of the experimental spectrum when acquiring the DEER data. Under these conditions, the resulting frequency distribution (Pake pattern) will always contain a contribution from the perpendicular component of the dipolar coupling, corresponding to the 'true' inter-copper coupling frequency [2]. Suppression of orientations near $\theta = 0°$ may contribute to the slight disagreement between experiment and simulations as evident in the main part of the manuscript (Figure 3), but does not affect the conclusions drawn in this study.

Supplementary Note 5: Additional EPR Data and Simulations

Verification of Exchange Coupling

To prove that the high frequency modulation in the DEER data for **P2**||**P2** is indeed due to exchange coupling between the two copper centers, additional DEER measurements were performed at a different spectrometer frequency and different concentration. The results of the measurements are shown in **Supplementary Figure 9**. If the modulation was due to nuclear ESEEM effects, the modulation frequency would need to change when performing the measurements at a different spectrometer frequency as the nuclear Larmor frequencies change. If the modulation was due to intermolecular interactions, the frequency should be affected by changing the sample concentration. Since the measured modulation frequency was found to be the same in all of these experiments, intramolecular interactions have to be at the origin of the modulation. For a dipolar modulation, the frequency is too high; especially since the Cu$\cdots$Cu distance in the system can be predicted quite accurately based on experimental data available for a similar ring system [3] and is expected to amount to roughly 2.4 nm. The modulation thus has to be caused by exchange coupling.

EPR Characterization of the Samples

The measured (raw) DEER data before any background-correction is shown for all three investigated samples in **Supplementary Figure 10** together with the Fourier transformations calculated from the

corresponding background-corrected traces shown in the main text. Maxima at roughly 3.5 MHz, 5.2 MHz and 13.1 MHz are detected for **X**||**X**, **P2**||**X** and **P2**||**P2**, respectively.

Additionally, Davies ^{14}N ENDOR measurements were performed on all samples at field positions corresponding to the $g_{\perp}$ (xy) and $g_{||}$ (z) orientations of the electronic **g**-tensor in order to get complementary information on the complex formation and consequently sample composition. If copper coordinates to axial nitrogen ligands, the hyperfine couplings of the nitrogen atoms within the porphyrin plane are reduced by roughly 3 MHz, leading to a shift of the whole spectrum to lower frequencies [4]. Consequently, it should be possible to judge from the ENDOR spectra whether complex formation is complete under the same conditions as applied in the DEER measurements (frozen toluene-d_8, 15 K). The composition of the sample can be analyzed and this information can then be used in the interpretation of the DEER traces regarding, for instance, background contributions and modulation depths. The data are shown in **Supplementary Figure 11** and reveal that in both samples, **X**||**X** and **P2**||**X**, a considerable amount of copper porphyrin units are not bound to an axial nitrogen ligand. The spectra are in-between those of **P2**||**P2**, where all copper units are bound to a nitrogen ligand, and $\mathbf{P6''_{Cu2}}$ (shown in the background in gray) where no copper units are bound to an axial ligand. Complex formation seems more 'complete' in the **P2**||**X** sample as compared to **X**||**X**, as was to be expected based on the UV-vis-NIR results.

The ENDOR results clearly confirm the conclusions from the UV-vis-NIR measurements, namely that the samples of **P2**||**X** and **X**||**X** are composed of several different species. However, most of these species will not influence the DEER measurements at all since they contain either one or no copper centers. Some species might lead to additional background contributions, but these contributions can be accounted for in the analysis and do not influence the dominating modulation frequency and its distribution on which the interpretations of the data in the main text were based. Aggregation can be excluded as a possible cause of background contributions to the DEER data, based on the data recorded at different concentrations presented in **Supplementary Figure 9**.

Sign of the Exchange Interaction

According to the definition of the dipolar coupling Hamiltonian

$$\hat{\mathcal{H}}_D = \frac{\mu_0 \gamma_A \gamma_B \hbar}{4\pi} \frac{1}{r_{AB}^3} \left(1 - 3\cos^2\theta\right) = \frac{\mu_0 g_A g_B \beta_e^2}{4\pi\hbar} \frac{1}{r_{AB}^3} \left(1 - 3\cos^2\theta\right) \tag{S9}$$

with

$$|\gamma_e| = \frac{g_e \beta_e}{\hbar} \tag{S10}$$

the D value corresponding to

$$D = \frac{\mu_0 g_A g_B \beta_e^2}{4\pi\hbar} \frac{1}{r_{AB}^3} \tag{S11}$$

has to be positive in all cases. Since we should be able to determine the relative sign of J from the simulations, the fact that D needs to be positive enables us to determine the absolute sign of J.

The simulations of the experimental data using the analytic expression for the determination of the exchange coupling have been performed using the following Hamiltonian

$$\hat{\mathcal{H}}_J = J\hat{\mathcal{S}}_A\hat{\mathcal{S}}_B \tag{S12}$$

A positive J value thus refers to antiferromagnetic coupling ($E_S < E_T$).

To a first approximation, the dominating modulation frequency detected in the experimental DEER trace should correspond to the absolute value of a combination frequency of D and J. Thus, even if D is positive, two solutions for J (one positive, one negative) leading to the same absolute value could be imagined. The dipolar coupling frequency in the studied systems has been determined to be $D = 3.6$ MHz. The best fit to the experimental data for **P2**||**P2** was obtained for a value of $J = 10.4$ MHz as shown in the main text, leading to a value of roughly 14 MHz for $D + J$. The same absolute value could be obtained for a J value of -17.6 MHz. The same reasoning for **P2**||**X** leads to an absolute value of 5.9 MHz which can be obtained for $J = 2.3$ MHz or $J = -9.5$ MHz.

However, we find that the simulations for the two cases of a positive or negative J value are significantly different for both samples (due to the Fresnel terms in the analytic expression of the dependence of the DEER trace on D and J), so that the sign of J can indeed be determined from a simulation of the experimental data. The simulations carried out for the case of a negative J (ferromagnetic coupling) are shown for both samples, **P2**||**P2** and **P2**||**X**, in **Supplementary Figure 12** and are compared to the simulations shown in the main text where $J > 0$. It was tested that also variations of these values by up to several MHz do not noticeably improve the goodness of fit. It can clearly be stated that the agreement is much worse as compared to the data presented in the main text (for positive J) demonstrating that the sign of J can be determined with confidence.

Discussion of the DEER Background

As was already mentioned briefly when discussing the UV-vis-NIR and ENDOR data of the EPR samples, incomplete complex formation leads to the presence of different species in the samples of **X**||**X** and **P2**||**X** and can lead to background contributions to the DEER data. Such additional contributions to the signal are only expected for species/complexes with more than one copper center and could account for most or all of the observed small mismatch between the relative modulation amplitudes (i.e. intensities) of experimental DEER data and the simulations presented in the main text. Since the amplitudes are not considered in the interpretation of the data, these contributions do not affect our conclusions, however it is desirable to understand the origin of these discrepancies.

The UV-vis-NIR and ENDOR data suggests that the differences in amplitude between experiment and simulations arise from contributions of at least a second species to the DEER data. In the case of $\mathbf{P3''_{Cu}}$, we saw that 2:1 complex formation is not complete if only 0.5 stoichiometric equivalents of **T6** are added. We do not expect any signal from unbound $\mathbf{P3''_{Cu}}$, nor from **T6** or the 1:1 complex $\mathbf{P3''_{Cu}} \cdot \mathbf{T6}$, but since it is known that Zn has a much higher affinity to bind to axial nitrogen ligands than Cu, more complicated complexes involving two **T6** templates and at least two $\mathbf{P3''_{Cu}}$ molecules could be imagined and could lead to the presence of various longer separation distances.

To demonstrate that a satisfactory simulation of the experimental data can be achieved by including a minor contribution accounting for different arrangements of **T6** and **P3**$''_{\mathbf{Cu}}$, a second component with a broad contribution centered at a longer distance was included in the simulation of the data for **X**||**X**. The center and width of this second component were allowed to vary until the best possible fit was obtained. The result of this simulation is shown in **Supplementary Figure 13** and the corresponding distributions in the distance and frequency domain are depicted in **Supplementary Figure 14**. The (small) remaining difference between experimental data and simulation might be a consequence of orientation selection.

The DEER trace of the **X**||**X** sample can well be reproduced by including longer distances in addition to the dominating modulation with a frequency of 3.6 MHz. The presence of longer distances causes an ‘uplift’ of the dipolar evolution trace at early times which cannot be reproduced by the simulation with just one component shown in the main text.

In the case of **P6**$''_{\mathbf{Cu2}}$, complex formation seems complete (at 298 K) based on the UV-vis-NIR titrations, however the ENDOR results (cf. **Supplementary Figure 11**) indicate the presence of a small amount of unbound copper porphyrin units. Since free **P6**$''_{\mathbf{Cu2}}$ is expected to contribute to the DEER data, this minor contribution might account for the amplitude differences observed between experiment and simulation for this sample. For reference, the DEER data (before background-correction) recorded for **P6**$''_{\mathbf{Cu2}}$ without template in toluene-d_8 is shown in **Supplementary Figure 15** in comparison to the data for **P2**||**X**.

P6$''_{\mathbf{Cu2}}$ is equivalent to **P2**||**X** without the **T6** template stabilizing the molecular geometry. The comparison of the corresponding DEER traces in **Supplementary Figure 15** clearly shows that any effect of J-coupling on the experimental DEER data is indiscernible in the linear **P6**$''_{\mathbf{Cu2}}$ compound, most likely due to a broad distribution of couplings owing to the relatively large flexibility of the structure as compared to **P2**||**X**.

Supplemenatry Note 6: DFT Calculations of the Exchange Interaction

The values of J measured in this work are at the limit of what can be calculated by DFT, although J-couplings of a similar magnitude have been calculated before, and the trends were consistent with experimental results [5].

In our case, DFT was able to reproduce the sign of J for **P2**||**P2** and **P2**||**X** and confirmed the increase in J for the two-path vs. the one-path structure. However, our calculations were inconclusive as to the magnitude of the increase, reflecting the inherent difficulties of using DFT to calculate such small energy differences.

We calculated J by comparing the energies of triplet and broken symmetry (singlet) wavefunctions, using Yamaguchis formula [6, 7]. From an initial converged triplet geometry and wavefunction, two methods were used to generate the singlet solution: Method (1): a broken symmetry guess at the triplet geometry was converged; Method (2): a further geometry optimization in the broken symmetry state was performed. The results of both calculations are summarized in the table below.

$$J = \frac{E_{BS} - E_T}{\langle S^2 \rangle_T - \langle S^2 \rangle_{BS}} \quad \text{(S13)}$$

where E_{BS} is the energy of the broken symmetry (singlet) state, E_T is the energy of the triplet state, and $\langle S^2 \rangle_{BS}$ and $\langle S^2 \rangle_T$ are expectation values of S^2 for the broken symmetry and triplet states.

	**P2‖P2**	**P2‖X**
Method (1)	−28 MHz	−12 MHz
Method (2)	−38 MHz	−6 MHz

The geometry optimizations employed a convergence criterion of 10^{-8} a.u.; SCF calculations employed a density convergence criterion of 10^{-12} and a 'superfine' integral grid. The stability of the final wavefunctions was checked, and converged geometries were confirmed as minima by the absence of imaginary frequencies in the vibrational analysis. For **P2‖X** a model of Cu−Zn−Zn−Cu **P4** was used, with a bend enforced by the use of constraints in the geometry optimization. In all structures, the solubilizing *meso*-aryl groups (Ar in **Figure 1**) were truncated to −H. All calculations used the B3LYP/6-31G* functional/basis set combination in Gaussian09/D.01.

Since the DFT calculations make use of the following Hamiltonian

$$\hat{\mathcal{H}}_J = -2J\,\hat{\mathcal{S}}_A\hat{\mathcal{S}}_B \quad \text{(S14)}$$

a negative J value indicates antiferromagnetic coupling ($E_S < E_T$), in agreement with the experimental data and simulations.

Supplementary References

[1] Cremers, J.; Richert, S.; Kondratuk, D. V.; Claridge, T. D. W.; Timmel, C. R.; Anderson, H. L. Nanorings with Copper(II) and Zinc(II) Centers: Forcing Copper Porphyrins to Bind Axial Ligands in Heterometallated Oligomers. *Chem. Sci.* **7**, 6961–6968 (2016).

[2] Bowen, A. M.; Tait, C. E.; Timmel, C. R.; Harmer, J. R. Orientation-Selective DEER Using Rigid Spin Labels, Cofactors, Metals, and Clusters. In *Structural Information from Spin-Labels and Intrinsic Paramagnetic Centres in the Biosciences*; Timmel, C. R., Harmer, J. R., Eds.; Structure and Bonding; Springer: Berlin Heidelberg, 2013; Vol. 152; Chapter 7, pp 283–327.

[3] Sprafke, J. K.; Kondratuk, D. V.; Wykes, M.; Thompson, A. L.; Hoffmann, M.; Drevinskas, R.; Chen, W.- H.; Yong, C. K.; Kärnbratt, J.; Bullock, J. E.; Malfois, M.; Wasielewski, M. R.; Albinsson, B.; Herz, L. M.; Zigmantas, D.; Beljonne, D. and Anderson, H. L. Belt-Shaped π-Systems: Relating Geometry to Electronic Structure in a Six- Porphyrin Nanoring. *J. Am. Chem. Soc.* **133**, 17262–17273 (2011).

[4] Richert, S.; Cremers, J.; Anderson, H. L.; Timmel, C. R. Exploring Template-Bound Dinuclear Copper Porphyrin Nanorings by EPR Spectroscopy. *Chem. Sci.* **7**, 6952–6960 (2016).

[5] Riplinger, C.; Kao, J. P. Y.; Rosen, G. M.; Kathirvelu, V.; Eaton, G. R.; Eaton, S. S.; Kutateladze, A.; Neese, F. Interaction of Radical Pairs Through-Bond and Through-Space: Scope and Limitations of the Point-Dipole Approximation in Electron Paramagnetic Resonance Spectroscopy. *J. Am. Chem. Soc.* **131**, 10092–10106 (2009).

[6] Yamaguchi, Yamaguchi, K.; Fukui, H.; Fueno, T. Molecular Orbital (MO) Theory for Magnetically Interacting Organic Compounds. Ab-initio MO Calculations of the Effective Exchange Integrals for Cyclophane-type Carbene Dimers. *Chem. Lett.* **4**, 625–628 (1986).

[7] Nishino, M.; Yamanaka, S.; Yoshioka, Y.; Yamaguchi, K. Theoretical Approaches to Direct Exchange Couplings between Divalent Chromium Ions in Naked Dimers, Tetramers, and Clusters. *J. Phys. Chem. A* **101**, 705–712 (1997).
